# The Importance of Cardiac Biomarkers on Remodelling After Myocardial Infarction

**DOI:** 10.4021/jocmr759w

**Published:** 2012-01-17

**Authors:** Ahmet Celik, Nihat Kalay, Omer Sahin, Mustafa Duran, Hasan Korkmaz, Mehmet Ali Kobat, Ertugrul Kurtoglu, Ali Dogan, Sabahattin Muhtaroglu, Oguzhan Baran, Mehmet Tugrul Inanc, Ibrahim Ozdogru, Abdurrahman Oguzhan, Ramazan Topsakal

**Affiliations:** a Department of Cardiology, Elazig Education and Research Hospital, Elazig, Turkey; b Department of Cardiology, Erciyes University Medical Faculty, Kayseri, Turkey; c Department of Cardiology, Kayseri Education and Research Hospital, Kayseri, Turkey; d Department of Biochemistry, Erciyes University Medical Faculty, Kayseri, Turkey

## Abstract

**Background:**

The purpose of this study is to evaluate the importance of tenascin-C ( TNC), N-terminal pro brain natriuretic peptide (NT-proBNP) and C-reactive protein (CRP) on LV remodelling after myocardial infarction (MI).

**Methods:**

Fifty-seven stable patients with subacute anterior MI who had total or subtotal occlusion in the infarct-related left anterior desending artery in coronary angiography were enrolled the study. 18 of patients who had total occlusion received only medical theraphy, 19 of patients who had total occlusion received successful PCI+ medical theraphy and 20 of patients who had subtotal occlusion received successful PCI+ medical theraphy. Left ventricular volumes and ejection fractions (EF) were measured with echocardiography. Serum TNC, NT-proBNP and CRP levels were measured at admission and a month after treatment.

**Results:**

There was significant increase in LV end-diastolic volume (LVEDV) and LV end-systolic volume (LVESV) baseline to follow-up in total-PCI group. Baseline to follow-up; a borderline significant increase was observed in LVEDV in the total-medical group. No significant difference was seen in LV volumes and EF in the subtotal-PCI group. NT-proBNP, TNC and CRP levels were decreased in all groups. The decrease in NT-proBNP and CRP values were significant in the total-medical and subtotal-PCI group but in the total-PCI group they were not significant. The decrease of TNC was significant in all groups but the lowest decrease was seen in the total-PCI group.

**Conclusion:**

TNC, NT-proBNP and CRP reflect LV remodelling in accordance with echocardiography after MI.

**Keywords:**

Tenascin-C; NT-pro BNP; CRP; Remodelling; Myocardial infarction

## Introduction

Fibrinolytic therapy or primary percutaneous coronary intervention (PCI) is early reperfusion strategies to treat ST segment elevation myocardial infarction (MI). However these strategies cannot be performed in about one third of patients because of late presentation [1]. Therefore, the management of patients experiencing late phase MI is an important clinical issue. Previous studies showed that PCI had no clinical benefit for patients with total occlusion of the infarct-related coronary artery [2-4]. Based on these studies we aimed to confirm the unfavorable effect of PCI on total occlusion after MI with cardiac biomarkers such as tenascin-C (TNC), N-terminal pro brain natriuretic peptide (NT-proBNP) and C-reactive protein (CRP). TNC is an extracellular matrix glycoprotein that is expressed in various cardiac pathological conditions, including; MI [5,6], myocarditis [7], hibernating myocardium and LV (LV) remodeling [8]. Brain natriuretic peptide (BNP) is a cardiac neurohormone that is secreted from the ventricular myocardium. It is secreted as a response to increased LV wall stress and is related with LV systolic dysfunction [9] and progressive remodeling after MI [10]. Persistent high plasma BNP levels after MI indicate LV remodeling and progressive heart failure. C- reactive protein (CRP) is a marker commonly used to show acute inflammatory response. It has also been used to demonstrate ventricular remodeling after acute MI [11].

In this study, we aimed to demonstrate the usefullness of TNC, NT-proBNP and CRP on LV remodeling after MI.

## Methods

### Study population

Fifty-seven patients with subacute anterior wall MI were enrolled in the study. Exclusion criteria included the following: patients who had received fibrinolytic therapy or who had PCI performed in the early stages of MI, any findings suggesting ongoing myocardial ischemia, angina at rest, NYHA class III or IV heart failure, shock, a serum creatinine concentration higher than 2.5 mg per deciliter, angiographically significant left main or three-vessel coronary artery disease, any significant stenosis in the right or circumflex coronary artery together with an LAD artery lesion, history of coronary artery disease, cardiac muscle disease, bundle branch block or atrial fibrillation, hemodynamic and electrical instability. Unsuccessful PCI was also an exclusion criterion in the groups to which PCI was applied.

Conventional coronary angiography was performed with Philips Integris 5000 equipment (Philips Medical Systems, Best, The Netherlands) in patients within 1 to 3 days after admission. After obtaining images by standard approaches, each angiogram was interpreted by two independent cardiologists. The coronary lesions were classified as total occlusions or subtotal occlusive lesions. The criterion for total occlusion of the LAD artery was absent antegrade flow, defined as a Thrombolysis in Myocardial Infarction (TIMI) flow grade of 0.

Patients were divided into three groups according to their angiographic characteristics and treatment options. The total-PCI group consisted of 18 patients with total occlusion in the LAD artery in whom PCI was performed together with medical therapy. The total-medical group consisted 19 patients with total occlusion in LAD and received only medical therapy. The subtotal-PCI group consisted 20 patients who had subtotal occlusion in the LAD artery in whom PCI was performed together with medical therapy. The patients in the total-PCI and subtotal-PCI groups were assigned to PCI with stent placement. Optimal medical therapy included aspirin, angiotensin converting enzyme inhibitors, beta-blockers, lipid lowering therapy, and clopidogrel.

In the total-PCI and subtotal-PCI groups, PCI was performed at 2 - 28 days after MI. Successful PCI was defined as an open artery with residual stenosis of less than 30% and a TIMI flow grade of 3. The study was approved by the local ethics committee. All the patients were informed about the study, and their written consent forms were obtained.

### Echocardiography

The Echocardiographies were performed by two cardiology specialist with Vivid 7 instruments (GE Medical Systems, Milwaukee, WI, USA), with a 2.5 MHz transducer and harmonic imaging in the cardiology department’s echocardiography laboratory. LV end diastolic (LVEDV) and end systolic volumes (LVESV) were measured at apical four chambers view. LV ejection fraction (LVEF) was assessed using the modified biplane Simpson’s method.

### Statistical analysis

Data are expressed as mean ± SD, or as a percentage. Comparisons between the groups were carried out using a One-way Anova test. To compare the change in measurements between baseline and at one month in each group, a Student’s paired test was used. SPSS 15.0 software was used for statistical analysis (Version 15, SPSS Inc., Chicago, IL, USA).

## Results

The demographic characteristics of the patients were similar in all groups (Table 1).

**Table 1:** Baseline Characteristics of All Patients in Three Groups

****	**Total-PCI Group (n: 18)**	**Total-Medical Group (n: 19)**	**Subtotal-PCI Group (n: 20)**	**P value**
Age	62 ± 12	65 ± 8	59 ± 12	0.2
Female (n, %)	5 (27)	6 (31)	6 (30)	0.9
DM (n, %)	8 (44)	9 (47)	4 (20)	0.1
HT (n, %)	9 (50)	10 (52)	6 (30)	0.3
Smoke (n, %)	11 (61)	10 (52)	12 (60)	0.8
CVD (n, %)	1 (5)	2 (10)	0	0.3
NYHA Class (n, %)				
I	7 (39)	8 (42)	9 (45)	0.9
II	11(61)	11(58)	11(55)	0.9
III- IV	0	0	0	
Blood Pressure(mmHg)				
Diastolic	82 ± 18	85 ± 15	75 ± 11	0.1
Systolic	133 ± 23	137 ± 28	125 ± 22	0.3
Creatinine (mg/dL)	1.0 ± 0.2	1.1 ± 0.4	0.9 ± 0.1	0.1
Lipid profile (mg/dL)				
LDL-C	120 ± 29	116 ± 43	118 ± 34	0.9
HDL-C	39 ± 9	41 ± 10	37 ± 8	0.4
T.CHOL	192 ± 46	183 ± 54	184 ± 42	0.8
TG	165 ± 131	129 ± 89	144 ± 80	0.5

Data expressed as mean ± SD, P < 0.05 was accepted as a statistically significant. DM: diabetes mellitus; HT: hypertension; CVD: cerebrovascular disease; LDL-C: low density lipoprotein; HDL-C: high density lipoprotein; T.CHOL: total cholesterol; TG: trigliseride

In the total-PCI and subtotal-PCI groups, the average of mean times from MI to intervention was 5 days.

The NT-proBNP, TNC and CRP levels were decreased in all groups at one month compared to baseline values. TNC levels were significantly decreased at one month in all three groups. NT-proBNP and CRP values were not significantly decreased in the total-PCI group but were significantly decreased in both the total-medical and subtotal-PCI groups at one month (Table 2).

**Table 2:** The Change of Cardiac Biomarkers at Baseline and First Month in All Groups

**Total- PCI Group (n = 18)**	**Baseline**	**First month**	**P value**
TENASCIN- C (ng/mL)	24 ± 12	14 ± 8	0.01
NT-PROBNP (pg/mL)	14311 ± 6962	11249 ± 5918	0.08
CRP (mg/dL)	24 ± 6	10 ± 2	0.07
Total-Medical Group (n = 19)			
TENASCIN-C (ng/mL)	21 ± 8	13 ± 4	< 0.001
NT-PROBNP (pg/mL)	12284 ± 7527	3661 ± 3973	< 0.001
CRP (mg/dL)	82 ± 12	8 ± 6	0.003
Subtotal-PCI Group (n = 20)			
TENASCIN-C (ng/mL)	22 ± 7	13 ± 5	< 0.001
NT-PROBNP (pg/mL)	15329 ± 6281	5305 ± 6141	< 0.001
CRP (mg/dL)	23 ± 4	5 ± 2	0.004

Data expressed as mean ± SD, P < 0.05 was accepted as a statistically significant. NT-proBNP: N- terminal natriuretic peptide; CRP: C-reaktive protein

In the total-PCI group, no significant difference was found between baseline and first month in LVEF. However, a significant increase was observed in LVEDV and LVESV (Table 3). No significant difference was found at follow-up comparing to the baseline values in LVESV, LVEDV and LVEF in the subtotal-PCI group (Table 3).

**Table 3:** The Change of LV Volumes and Functions Baseline to First Month in All Groups

**Total- PCI Group (n = 18)**	**Baseline**	**First month**	**P value**
LVESV (mL/mm^2^)	59 ± 27	68 ± 26	0.03
LVEDV (mL/mm^2^)	98 ± 35	114 ± 35	0.01
LVEF (%)	39 ± 8	40 ± 8	0.1
Total-Medical Group (n = 19)			
LVESV (mL/mm^2^)	75 ± 23	82 ± 25	0.1
LVEDV (mL/mm^2^)	113 ± 27	126 ± 29	0.05
LVEF (%)	35 ± 9	36 ± 9	0.2
Subtotal-PCI Group (n = 20)			
LVESV (mL/mm^2^)	66 ± 30	68 ± 32	0.4
LVEDV (mL/mm^2^)	113 ± 40	115 ± 38	0.5
LVEF (%)	42 ± 9	42 ± 9	0.3

Data expressed as mean ± SD, P < 0.05 was accepted as a statistically significant. LVEDV: Left ventricular enddiastolic volume; LVESV: Left ventricular endsystolic volume; LVEF: Left ventricular ejection fraction

Comparing to the baseline values; LVESV and LVEF were not significantly changed at the first month in the total-medical group (Table 3). A borderline significant increase was observed in LVEDV at the first month compared to baseline measurement (LVEDV: 113 ± 6 mL versus 126 ± 6 mL, P = 0.05).

**Figure 1:**
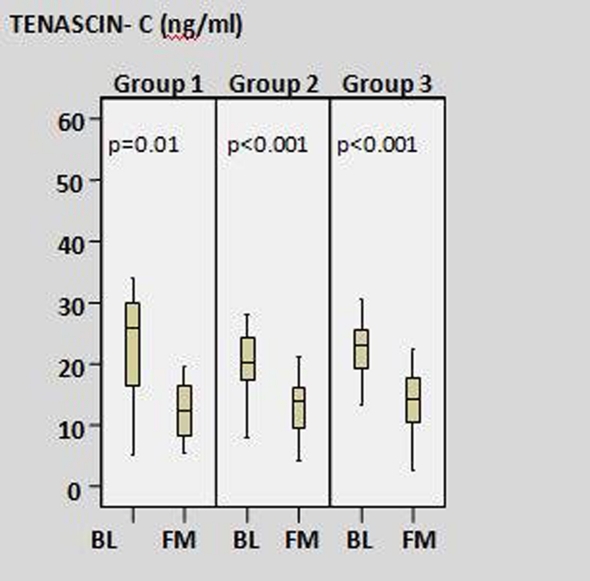
The change of tenascin-C levels between baseline and first month in all study groups. (Group 1: Total-PCI group; Group 2: Total-medical group; Group 3: Subtotal-PCI group; BL: baseline; FM: first month).

Figure 1 shows the change of tenascin-C levels in the three groups. The lowest decrease in the levels of TNC were seen in the total-PCI group at one month after MI.

**Figure 2:**
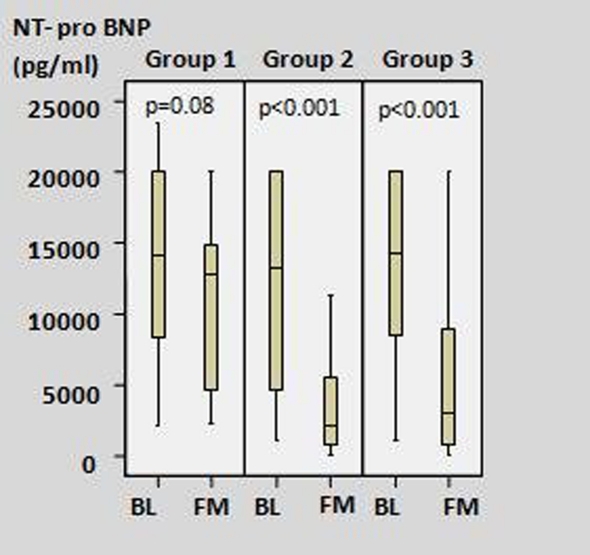
The change of NT-proBNP levels between baseline and first month in all study groups. (Group 1: Total-PCI group; Group 2: Total-medical group; Group 3: Subtotal-PCI group; BL: baseline; FM: first month)

Figure 2 shows the change in NT-pro BNP levels in the three groups. At first month after MI, the lowest decrease NT-pro BNP levels was seen in the total-PCI group.

## Discussion

Using PCI to treat infarct-related artery occlusion in stable patients is still a controversial issue. We assessed the effect of PCI and optimal medical therapy in patients with total or subtotal occlusion in an infarct-related LAD artery. Our present results showed that PCI applied to total occlusion in an infarct-related LAD artery caused higher LV remodeling when compared to medical therapy for total occlusion and PCI for total occlusion. These results emphasize the importance of avoiding invasive intervention in stable patients with total occlusion in the infarct-related LAD artery. For many years, the open artery hypothesis was defended as a treatment that would improve LV functions and reduce LV remodeling after late reperfusion [12,13]. However, in recent years, large randomized studies demonstrated no clinical benefit from percutaneous coronary intervention for a totally occluded infarct-related artery in asymptomatic patients who had hemodynamically and electrically stable clinical findings and who did not have evidence of severe ischemia [2,4,14,15]. Based on these results, current guidelines do not recommend PCI for a totally occluded infarct-related artery in asymptomatic patients [16].

On the other hand, we analyzed the effect of cardiac biomarkers on LV remodeling in stable patients after MI. Thus, we aimed to compare the biochemical data of remodeling in patients in whom PCI was applied for the treatment of a total or subtotal occlusion infarct-related artery after MI.

Tenascin-C is used as a marker that shows LV remodeling and long-term prognosis after MI [8]. Increased TNC levels had a relationship with complexity of coronary lesion after MI [17], severity of heart failure and LV remodelling in dilated cardiomyopathy [18]. CRP and neurohormones, which were secreted from cardiomyocytes like atrial natriuretic peptides (ANP) and brain natriuretic peptides (BNP), are also used to show LV remodeling [10,11]. We aimed to show LV remodeling with biomarkers such as TNC, NT-pro BNP and CRP. Compared to the baseline values, NT pro-BNP, TNC and CRP levels were decreased in all groups at one month. In the total- PCI group the decrease of NT-proBNP and CRP values was not statistically significant but the TNC values were significantly decreased. Hovewer, the rate of decrease in TNC levels was lower in the total-PCI group than in the other groups. In both the total-medical and subtotal-medical groups, there were significant decreases in the TNC, NT-proBNP and CRP values at one month.

Like the values of LV volumes and EF on echocardiography; serum levels of TNC, NT-proBNP and CRP showed the LV remodelling after MI. This study is very important to demonstrate the LV remodelling with cardiac biomarkers.

Our study’s most important limitation was the small number of patients. Follow-up time was only one month. Therefore, large and long-term follow-up studies are needed.
